# Dieulafoy’s Lesion: A Rare and Elusive Cause of Massive Upper Gastrointestinal Bleeding Managed With Endoscopic Therapy

**DOI:** 10.7759/cureus.77206

**Published:** 2025-01-09

**Authors:** Mustafa M Alkhanjar, Hawra A Hasan, Shaikha H Albanna, Rawah S Almutawa, Njood Alsudairy

**Affiliations:** 1 College of Medicine, Southeast University, Nanjing, CHN; 2 General Practice, Dammam Medical Complex, Dammam, SAU; 3 College of Medicine, Mansoura University, Mansoura, EGY; 4 General Practice, Ibn Al-Nafees Hospital, Manama, BHR; 5 Radiology, Jeddah Second Health Cluster, Jeddah, SAU

**Keywords:** computed tomography angiography, diagnosis, dieulafoy’s lesion, endoscopy, epinephrine injection, gastrointestinal hemorrhage, hematemesis, thermal coagulation, upper gastrointestinal bleeding, vascular anomaly

## Abstract

A Dieulafoy’s lesion is a rare vascular anomaly that can cause massive upper gastrointestinal bleeding. It often presents with hematemesis and requires prompt diagnosis and intervention. We report a case of a 54-year-old male patient with hypertension and diabetes mellitus who presented to the emergency department with hematemesis. After the initial resuscitation, an esophagogastroduodenoscopy revealed a pulsatile submucosal artery in the proximal stomach, consistent with a Dieulafoy’s lesion. No evidence of peptic ulcer disease, gastric varices, or malignancy was noted. Endoscopic hemostasis was achieved using an epinephrine injection followed by thermal coagulation. The patient’s hospital course was uncomplicated, with no further bleeding episodes. Computed tomography angiography confirmed the diagnosis and ruled out other potential sources of bleeding. The patient was discharged on a proton pump inhibitor regimen and scheduled for a follow-up endoscopy in six weeks, which showed complete mucosal healing. A Dieulafoy’s lesion remains a challenging diagnosis due to its subtle presentation and requires a high index of suspicion, especially in patients with unexplained gastrointestinal bleeding. Endoscopic therapy is highly effective in managing this condition and, when promptly addressed, results in excellent outcomes. This case highlights the importance of early recognition and intervention, contributing to the growing body of evidence supporting the role of endoscopy in the management of Dieulafoy’s lesion.

## Introduction

A Dieulafoy’s lesion is a rare but potentially life-threatening cause of upper gastrointestinal bleeding, accounting for approximately 1-2% of all cases [1,2]. First described by the French surgeon Georges Dieulafoy in 1898, this condition is characterized by an abnormally large, tortuous submucosal artery that erodes through the overlying mucosa without an associated ulcer or predisposing mucosal lesion. Most commonly found in the proximal stomach along the lesser curvature, Dieulafoy’s lesions can also occur in other areas of the gastrointestinal tract, including the duodenum, jejunum, colon, and rectum [1-3].

The clinical presentation is typically acute and dramatic, with patients experiencing massive hematemesis, melena, or hematochezia. Due to the rarity of this condition and the absence of typical ulcerative pathology, a Dieulafoy’s lesion often presents as a diagnostic challenge, especially in the setting of recurrent or obscure gastrointestinal bleeding [3,4]. Diagnostic modalities such as esophagogastroduodenoscopy and advanced imaging techniques, including computed tomography (CT) angiography, play a pivotal role in identifying the lesion [1,2].

Endoscopic therapy remains the cornerstone of treatment, with various techniques such as an epinephrine injection, thermal coagulation, or endoclipping being highly effective in achieving hemostasis. Early recognition and management are critical for preventing the significant morbidity and mortality associated with this underdiagnosed vascular anomaly.

## Case presentation

A 54-year-old male patient with a history of hypertension and type 2 diabetes mellitus presented to the emergency department with a chief complaint of hematemesis. He reported three episodes of vomiting bright red blood within the past 24 hours. The patient denied any history of melena, abdominal pain, or significant weight loss. He also denied any recent use of nonsteroidal anti-inflammatory drugs (NSAIDs), anticoagulants, or consumption of alcohol. There was no prior history of gastrointestinal bleeding or peptic ulcer disease. His family and social history were unremarkable.

On initial physical examination, the patient appeared pale but was alert and oriented. His vital signs revealed tachycardia with a heart rate of 112 beats per minute and borderline hypotension with a blood pressure of 95/60 mmHg. Capillary refill was delayed at four seconds. There was no evidence of jaundice, abdominal distension, or peritoneal signs. On digital rectal examination, his stool was negative for occult blood.

His laboratory workup revealed a hemoglobin level of 8.2 g/dL (reference range: 13.5-17.5 g/dL), indicating significant anemia. The platelet count, prothrombin time, and international normalized ratio were within normal limits. Serum creatinine and liver function tests were unremarkable. A nasogastric tube was inserted, and a lavage yielded fresh blood, confirming ongoing upper gastrointestinal bleeding.

Initial resuscitation included intravenous fluids and two units of packed red blood cells. The patient underwent emergent esophagogastroduodenoscopy, which revealed a pulsatile vessel in the submucosa of the proximal stomach, approximately 2 cm distal to the gastroesophageal junction, with active arterial bleeding. The surrounding gastric mucosa appeared normal without evidence of ulceration, varices, or malignancy. These findings were consistent with a Dieulafoy’s lesion. Endoscopic hemostasis was achieved using an epinephrine injection followed by thermal coagulation. Hemostasis was confirmed at the end of the procedure.

**Figure 1:**
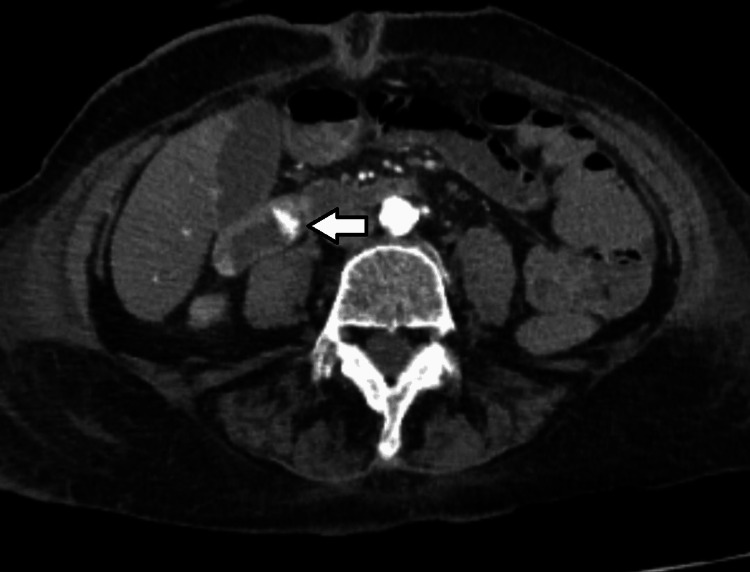
Axial CT of the abdomen Selected axial CT image of the abdomen showing a hyperenhancement focus within the duodenum (arrow) in the arterial phase.

A CT angiography of the abdomen was performed to evaluate for other potential sources of bleeding and to further characterize the vascular anatomy of the lesion. The imaging demonstrated a prominent submucosal artery in the proximal stomach without evidence of additional vascular malformations or pathology. The differential diagnosis included other vascular anomalies such as arteriovenous malformations, gastric varices, and aortoenteric fistula. However, the clinical and imaging findings supported the diagnosis of a Dieulafoy’s lesion (Figure 1).

Following endoscopic intervention, the patient was monitored in the intensive care unit for 48 hours. He remained hemodynamically stable with no further episodes of bleeding. His repeat hemoglobin levels stabilized at 10.4 g/dL, and serial nasogastric aspirates were clear. The patient was gradually transitioned from IV fluids to an oral diet, which he tolerated well. He was discharged on day five of hospitalization with instructions to avoid NSAIDs and to follow a proton pump inhibitor regimen for mucosal protection. 

## Discussion

A Dieulafoy’s lesion is a rare but significant cause of massive gastrointestinal bleeding. This case highlights several key aspects of its clinical presentation, diagnosis, and management. The patient’s abrupt onset of hematemesis without prior symptoms or risk factors underscores the characteristic presentation of Dieulafoy’s lesion as a source of acute, severe, and sometimes recurrent upper gastrointestinal bleeding. Consistent with the literature, the lesion in this case was located in the proximal stomach along the lesser curvature, which is reported as the most common site, accounting for approximately 70% of cases [1-4].

The diagnostic challenge posed by a Dieulafoy’s lesion stems from its subtle endoscopic appearance. Unlike peptic ulcers or varices, the mucosa surrounding the lesion appears unremarkable, making it easy to overlook during routine endoscopy. In this case, the identification of a pulsatile submucosal vessel during emergent esophagogastroduodenoscopy underscores the importance of meticulous examination during endoscopy, especially in patients with unexplained upper gastrointestinal bleeding. Advanced imaging, such as CT angiography, further supported the diagnosis by revealing a prominent submucosal artery without additional pathology. Studies have shown that CT angiography is particularly valuable in cases where endoscopic findings are inconclusive or when there is a suspicion of multiple bleeding sources [4,5].

The successful management of this patient with an endoscopic epinephrine injection and thermal coagulation reflects the efficacy of endoscopic techniques in controlling bleeding from Dieulafoy’s lesions. Current guidelines recommend endoscopic therapy as the first-line treatment, with reported success rates exceeding 90%. Various endoscopic modalities, including endoclipping, band ligation, and hemostatic sprays, have been described in the literature. In cases where endoscopic therapy fails or rebleeding occurs, angiographic embolization or surgical intervention may be required. Our case, however, demonstrated no rebleeding during hospitalization or follow-up, highlighting the durability of endoscopic management in appropriately-selected cases [2-6].

The recurrence of bleeding after initial treatment is reported in up to 15% of cases, often necessitating repeated endoscopic intervention. The absence of recurrence in this patient may be attributed to the thorough endoscopic therapy and the lack of significant comorbidities or predisposing factors, such as chronic liver disease or anticoagulant use [2,3]. Proton pump inhibitor therapy, as used in this case, is often prescribed post-procedure to reduce gastric acid exposure and promote mucosal healing, although its role in preventing recurrence of bleeding specific to the Dieulafoy’s lesion is not well established [1,4].

On a broader scale, Dieulafoy’s lesions serve as an important reminder of the diverse etiologies of gastrointestinal bleeding. While rare, it must be considered in the differential diagnosis, particularly in cases of massive or recurrent bleeding with normal mucosal findings. Advances in endoscopic and radiological techniques have significantly improved diagnostic accuracy and therapeutic outcomes, but continued awareness and clinical vigilance are essential for optimal patient care. This case contributes to the growing body of evidence supporting the efficacy and safety of endoscopic management for this unique vascular anomaly.

## Conclusions

A Dieulafoy’s lesion, though rare, is a critical consideration in cases with acute and massive gastrointestinal bleeding. Prompt recognition through meticulous endoscopic examination and advanced imaging is essential for accurate diagnosis. This case highlights the effectiveness of endoscopic intervention, specifically epinephrine injection and thermal coagulation, in achieving hemostasis and preventing recurrence. The patient’s favorable outcome underscores the importance of timely management and close follow-up to ensure resolution. As advancements in diagnostic and therapeutic modalities continue to evolve, early identification and treatment of vascular anomalies like Dieulafoy’s lesions will remain pivotal in reducing associated morbidity and mortality.

## References

[1] Baxter M, Aly EH (2010). Dieulafoy's lesion: current trends in diagnosis and management. Ann R Coll Surg Engl.

[2] Qasim A, Schmidt P, Bhatt T, Itare V, Ihimoyan A, Khaja M, Kandhi S (2023). Dieulafoy's lesion of the duodenum: a rare and fatal cause of gastrointestinal bleed. Cureus.

[3] Kusnik A, Mostafa MR, Sharma RP, Chodos A (2023). Dieulafoy lesion: scope it until you find it. Cureus.

[4] Wang Y, Bansal P, Li S, Iqbal Z, Cheryala M, Abougergi MS (2021). Dieulafoy's lesion of the upper GI tract: a comprehensive nationwide database analysis. Gastrointest Endosc.

[5] Joarder AI, Faruque MS, Nur-E-Elahi M (2014). Dieulafoy's lesion: an overview. Mymensingh Med J.

[6] Nakamura M, Yamamura T, Maeda K (2023). Clinical characteristics of Dieulafoy's lesion in the small bowel diagnosed and treated by double-balloon endoscopy. BMC Gastroenterol.

